# PRPF overexpression induces drug resistance through actin cytoskeleton rearrangement and epithelial-mesenchymal transition

**DOI:** 10.18632/oncotarget.17855

**Published:** 2017-05-15

**Authors:** Salman Ul Islam, Adeeb Shehzad, Jong Kyung Sonn, Young Sup Lee

**Affiliations:** ^1^ School of Life Sciences, College of Natural Sciences, Kyungpook National University, Daegu, Korea; ^2^ Department of Biomedical Engineering and Sciences, School of Mechanical and Manufacturing Engineering, National University of Sciences and Technology, Islamabad, Pakistan; ^3^ Department of Biology, College of Natural Sciences, Kyungpook National University, Daegu, Korea

**Keywords:** PRPF, resveratrol, actin filament, Rho family, EMT

## Abstract

Pre-mRNA processing factor (PRPF) 4B kinase belongs to the CDK-like kinase family, and is involved in pre-mRNA splicing, and in signal transduction. In this study, we observed that PRPF overexpression decreased the intracellular levels of reactive oxygen species, and inhibited resveratrol-induced apoptosis by activating the cell survival signaling proteins NFκB, ERK, and c-MYC in HCT116 human colon cancer cells. PRPF overexpression altered cellular morphology, and rearranged the actin cytoskeleton, by regulating the activity of Rho family proteins. Moreover, it decreased the activity of RhoA, but increased the expression of Rac1. In addition, PRPF triggered the epithelial-mesenchymal transition (EMT), and decreased the invasiveness of HCT116, PC3 human prostate, and B16-F10 melanoma cells. The loss of E-cadherin, a hallmark of EMT, was observed in HCT116 cells overexpressing PRPF. Taken together, these results indicate that PRPF blocks the apoptotic effects of resveratrol by activating cell survival signaling pathways, rearranging the actin cytoskeleton, and inducing EMT. The elucidation of the mechanisms that underlie anticancer drug resistance and the anti-apoptosis effect of PRPF may provide a therapeutic basis for inhibiting tumor growth and preventing metastasis in various cancers.

## INTRODUCTION

Pre-mRNA processing factor (PRPF), first identified in *Schizosaccharomyces pombe*, is a protein involved in pre-mRNA splicing [1]. Human *PRPF* encodes a 1,007-amino acid protein containing an N-terminal 340-amino acid Arg/Ser-rich domain, that is commonly found in pre-mRNA splicing factors [2]. The catalytic domain of PRPF shares homology with cyclin-dependent kinases and mitogen-activated protein kinases [3]. Mutations in PRPF result in pre-mRNA accumulation and in an impaired G1/S transition during the cell cycle [4]. Studies on PRPF have demonstrated its diverse effects on kinases, transcription factors, chromatin remodeling factors, spindle checkpoint proteins, and cancer cell growth [2, 5–7]. In support of a role for PRPF in pre-mRNA splicing, it has been reported that PRP6, SWAP, and PNN interact with PRPF, and PRPF was shown to be a U5 snRNP-associated kinase [8]. Moreover, PRPF was also reported to interact with BRG1 and N-CoR, two components of the nuclear hormone co-activator and co-repressor complexes. PRPF, in association with the U5 snRNP and N-CoR deacetylase complex, has been suggested to coordinate pre-mRNA splicing and the chromatin remodeling events involved in transcriptional regulation [8]. In particular, PRPF6, a member of the small ribonucleoprotein complex, was shown to promote colon cancer proliferation [9]. Recent studies reported that changes in the pattern of various alternative splicing events play prominent roles in colon cancer carcinogenesis and tumorigenesis [10–11]. Different splicing patterns have been observed for several alternative splicing events in colon cancers when compared to the patterns in normal tissues; among these, the inclusion of the *Rac1* 3b exon is the most noteworthy, because it specifically contributes to colon tumorigenesis through genomic instability and EMT [12–13].

EMT is a process by which epithelial cells lose their junctions, reorganize their cytoskeleton, and undergo cell signaling changes that define cell shape and lead to the reprogramming of gene expression [14–15]. During EMT, solid tumors gain metastatic characteristics. Various factors contribute to the development of anticancer drug resistance. However, all of these factors depend on the metastatic grade of the tumor, which can be defined by the level of differentiation and the degree of EMT [16–17]. EMT is integral to normal development. Nevertheless, its underlying processes are reactivated in fibrosis and during cancer progression. In addition, the suppression of EMT in cells can increase their sensitivity to anticancer drugs [18]. Therefore, the characterization of EMT and the identification of modulators of EMT-related molecules could potentially lead to new cancer treatments.

Previously, it was reported that PRPF is involved in reversing curcumin-induced cell death in human colon cancer cell lines [19]. PRPF also inhibited radiation-mediated cell death in colon cancer and in melanoma cells that were pre-sensitized with curcumin, possibly through the inhibition of the generation of reactive oxygen species (ROS), and the activation of the antioxidant enzyme system [20]. These studies represent a considerable attempt to elucidate the molecular mechanism of PRPF; however, its exact mode of action remains unclear. In the current study, we showed that PRPF overexpression in HCT116 cells inhibited resveratrol-induced apoptosis, and altered cell morphology, which was related to the rearrangement of the actin cytoskeleton. PRPF overexpression also inhibited RhoA activity and increased Rac1 expression, which led to anticancer drug resistance. Importantly, these findings advance our understanding of anticancer drug resistance in colon cancer.

## RESULTS

### PRPF inhibits resveratrol-induced apoptosis

**Figure 1:**
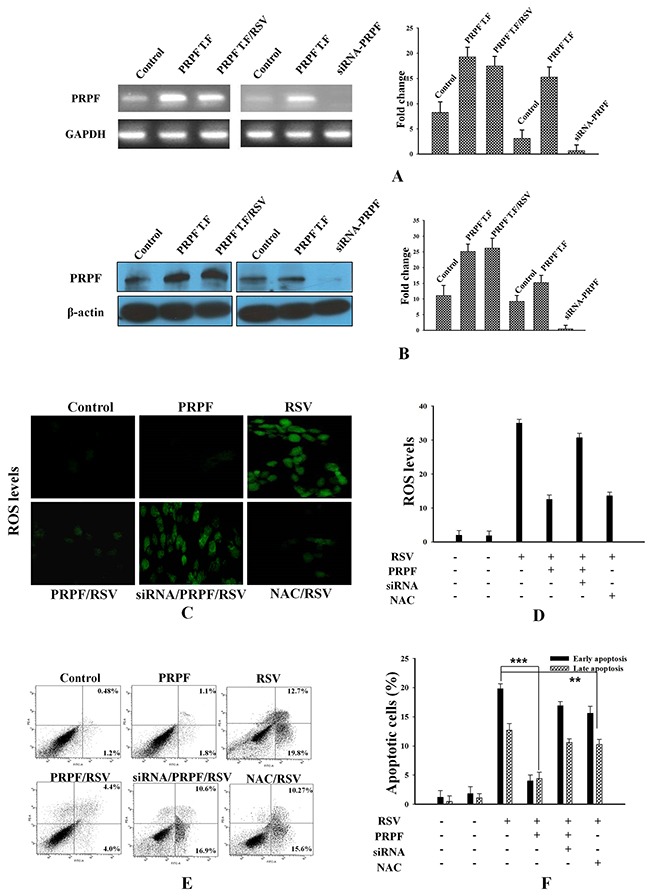
PRPF overexpression inhibits resveratrol-induced apoptosis **(A)** PRPF mRNA levels **(B)** PRPF protein levels. GAPDH is used as a loading control. **(C, D)** HCT116 cells transfected with a PRPF plasmid or siRNA-PRPF, and incubated with or without 10 μM resveratrol (RSV) for 24 h. The cells were incubated with the DCFHDA dye for 20 min to measure ROS. Probe accumulation was measured in triplicate based on increases in emission at a wavelength of 530 nm. ROS levels are expressed as the ratio of the mean intensity of the sample to the mean intensity in the control cells. **(E, F)** Parental and transfected HCT116 cells used in an annexin-V/propidium iodide assay and analyzed by flow cytometry to determine the levels of apoptosis. Data were collected from three independent experiments. **P < 0.01, ***P < 0.001.

It has been reported that resveratrol induces apoptosis in colon cancer cells by modulating multiple signaling pathways [21–22]. To investigate the inhibitory role of PRPF against resveratrol-induced apoptosis in HCT116 cells, these cells were transiently transfected with a PRPF expression construct, and then incubated with resveratrol for various periods of time. PRPF overexpression was confirmed both at the mRNA and protein levels by RT-PCR and western blotting, respectively (Figure 1A, B). We found that resveratrol did not affect PRPF expression at the mRNA and protein levels (Figure 1A, B). For PRPF knockdown, we used a pool of three target-specific siRNAs (PRPF-siRNA) 19-25 nucleotides in length, that downregulated PRPF expression at both the mRNA and protein levels (Figure 1A, B). To examine the effect of PRPF on resveratrol-induced ROS generation, HCT116 cells were treated with resveratrol, and incubated with a specific cell-permeable fluorescent dye, DCFHDA. DCFHDA is de-esterified and converted to the highly fluorescent 2′,7′-dichlorofluorescein upon oxidation. In resveratrol-treated cells, a considerable increase in 2′,7′-dichlorofluorescein fluorescence was observed, and PRPF overexpression prevented resveratrol-induced ROS generation (Figure 1C, D). PRPF-siRNA transfection restored resveratrol-induced ROS generation, and the pre-treatment with N-acetylcysteine (NAC), an oxidant scavenger, confirmed the involvement of the ROS pathway in resveratrol-induced apoptosis of HCT116 cells (Figure 1C, D). To further address whether PRPF inhibits resveratrol-induced apoptosis, an annexin V/propidium iodide (PI) apoptosis assay and flow cytometry were carried out. As shown in Figure 1E, F, resveratrol treatment resulted in 19.8% of cells in early apoptosis, and 12.7% cells in late apoptosis, compared to the respective percentages in untreated HCT116 cells, where cells found in early and late apoptosis represented less than 2%. PRPF overexpression signi-ficantly reduced resveratrol-induced apoptosis, and brought the percentages of cells in early and late apoptosis to 4.0% and 4.4%, respectively (Figure 1E, F). However, following concomitant transfection with a PRPF-expression plasmid and siRNA-PRPF, 16.9% of cells were observed in early apoptosis, which is close to the levels observed in resveratrol-treated HCT116 cells (Figure 1E, F). Similar results were observed after blocking ROS generation with N-acetylcysteine, an oxidant scavenger, before resveratrol exposure (Figure 1E, F). Collectively, these findings suggest that PRPF inhibits cell death, in part by blocking the resveratrol-induced generation of ROS.

### NFκB, ERK, and c-MYC mediate the anti-apoptotic effects of PRPF

**Figure 2:**
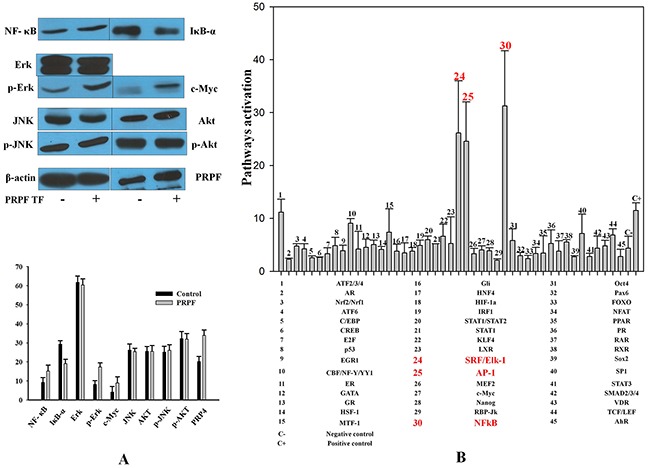
Resveratrol-induced cell death is inhibited by PRPF through activation of cell survival pathways **(A)** Western blotting of untransfected and PRPF-transfected HCT116 cell lysates to assess NFκB, IκBα, AKT, and c-MYC levels. Actin is used as a loading control. **(B)** Cignal 45-Pathway Reporter Array data in HCT116 cells upon PRPF overexpression. The experiment was performed in triplicate, and data represent the means ± SD.

To further elucidate the anti-apoptotic effects of PRPF, we analyzed the changes in various cell survival regulatory proteins, following PRPF overexpression. Following transfection with a PRPF-expression plasmid in HCT116 cells, the protein level of NFκB increased, and the expression of IκBα decreased (Figure 2A). PRPF overexpression in HCT116 cells also increased ERK phosphorylation, and significantly upregulated c-MYC expression. In contrast, PRPF overexpression did not affect the phosphorylation of AKT or JNK (Figure 2A). To determine the downstream targets of PRPF, we analyzed 45 signaling pathways using the Cignal 45-Pathway Reporter Array (Qiagen), and found that ELK-1/SRF, AP-1, and NFκB were significantly upregulated upon PRPF overexpression (Figure 2B). These results suggest that PRPF may inhibit resveratrol-induced cellular apoptosis by activating cell survival pathways such as the NFκB, ERK, and c-MYC pathways.

### PRPF remodels the actin cytoskeleton by regulating Rho family proteins

**Figure 3:**
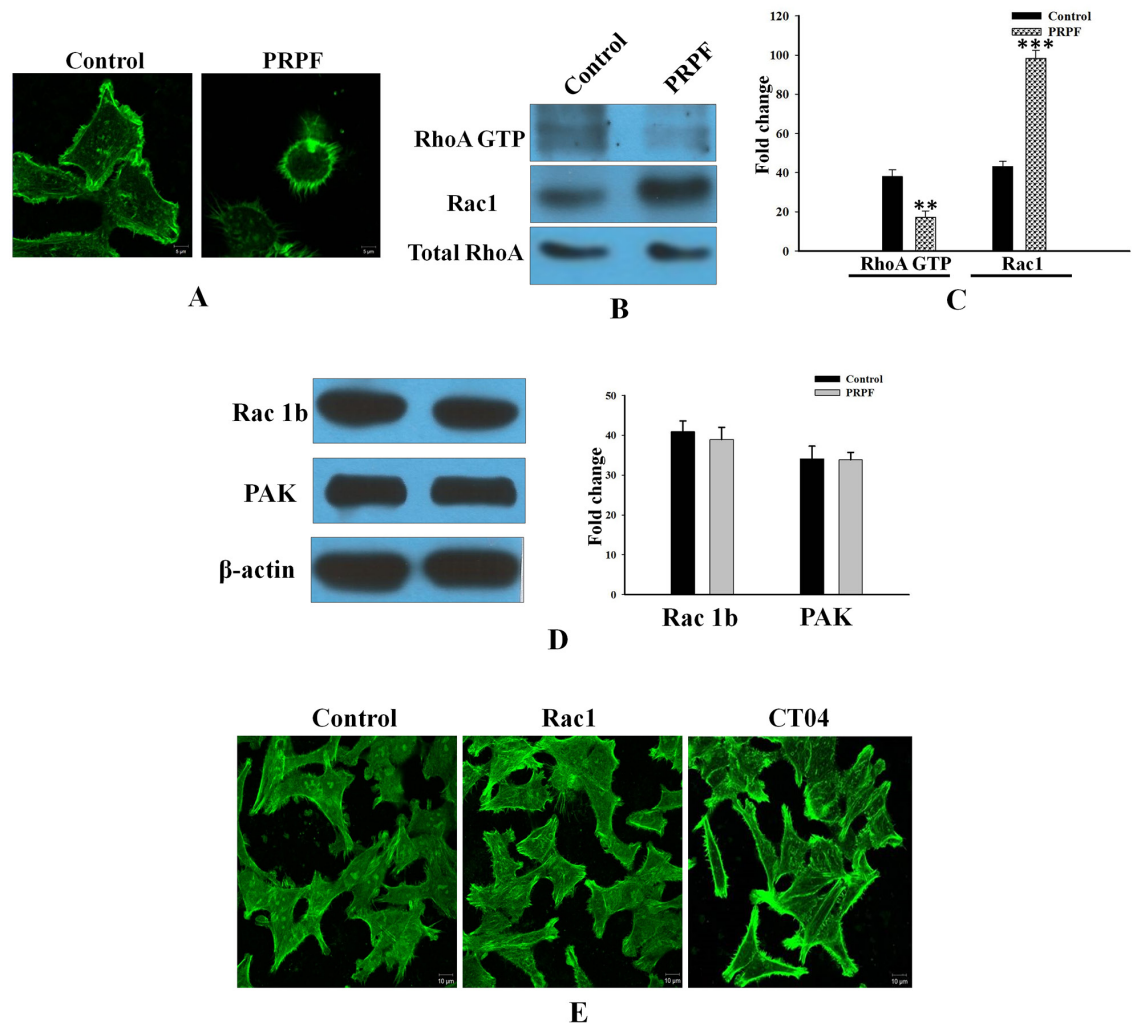
Resveratrol-induced cell death is inhibited by PRPF through activation of cell survival pathways **(A)** HCT116 cells transfected with a PRPF-expression plasmid, stained with phalloidin, and observed under a confocal laser microscope at 1000X magnification. **(B, C)** RhoA activity (in 400 μg of total cell lysate) detected on a 12% SDS-PAGE gel. Active RhoA is normalized to total RhoA, and the experiment is performed in duplicate. Data represent means ± SD. **P < 0.01; ***P < 0.001. **(D)** Western blotting using anti-Rac1B and anti-PAK antibodies. Actin is used as a loading control. **(E)** F-actin phalloidin staining in HCT116 cells transfected with a Rac1-expression plasmid, and incubated with the CT04 (1 μg/mL) RhoA inhibitor.

In addition to preventing apoptotic cell death, PRPF overexpression in HCT116 cells changed their morphology from an aggregated, flattened shape to a dispersed, round shape (Figure 3A). To assess whether the effects of PRPF overexpression were associated with modifications of the actin cytoskeleton, we stained actin filaments with Alexa Fluor 488-conjugated phalloidin, and visualized the actin filaments by fluorescence microscopy. While stress fibers were formed in the cytoplasm of the parental colon cancer cells, these fibers were absent in PRPF-overexpressing cells (Figure 3A). Actin microfilaments are the major structural filament of the cytoskeleton that performs key functions in internal organization and morphological changes [23–24]. It was reported that Rho family proteins are key regulators of actin cytoskeleton rearrangements. To examine whether RhoA is activated in PRPF-overexpressing cells, we performed a pull-down assay. GST-Rhotekin-RBD was incubated with the cell lysate to isolate GTP-loaded RhoA. The activated Rho GTPase bound to the GST fusion protein in the lysate was pulled down with colored glutathione-sepharose beads, and analyzed by western blotting. This showed that PRPF overexpression significantly decreased RhoA activity (Figure 3B, C).

It has been reported that another Rho family protein, Rac1, functions with RhoA in a mutually antagonistic manner [25]. Rac1 drives mesenchymal cell migration by stimulating the formation of actin-rich membrane extensions such as lamellipodia [26]. Control cancer cells displayed low Rac1 levels, whereas PRPF-transfected cells showed significantly elevated (2.5-fold increased) Rac1 levels. (Figure 3B, C). However, a well-known Rac1 effector, the p21 activated kinase (PAK), was expressed at about the same level in parental and PRPF-overexpressing HCT116 cells (Figure 3D). Unlike Rac1, the Rac1B splice variant was highly expressed in both control and PRPF-overexpressing HCT116 cells. Although Rac1B is upregulated in various malignant tumor cells, including HCT116, PC3, and B16-F10 cells, only low levels of Rac1B protein were detected in HCT-15 colon cancer cells. However, we found that in HCT-15 colorectal cells, Rac1B expression was significantly induced by transient transfection with a PRPF-expression plasmid (Supplemental Figure 1A).

To further investigate the molecular events underlying cytoskeletal dynamics in HCT116 cells, we inhibited Rho activity with the CT04 Rho inhibitor or overexpressed Rac1 using a Rac1 expression plasmid. Confocal images revealed that transfection with PRPF induced morphological and cytoskeletal changes in HCT116 cells; transfected cells appeared rounded, and actin stress fibers disappeared (Figure 3A). Consistent with these results, the number of actin fibers decreased when Rac1 was overexpressed (Figure 3E). In addition, CT04 treatment almost completely eliminated stress fibers in HCT116 cells, confirming the involvement of PRPF in actin cytoskeleton rearrangements (Figure 3E). These findings suggest that PRPF may remodel the actin cytoskeleton by regulating Rac1 and RhoA.

### Modulation of the Rho family inhibits resveratrol-induced apoptosis in cancer cell lines

**Figure 4:**
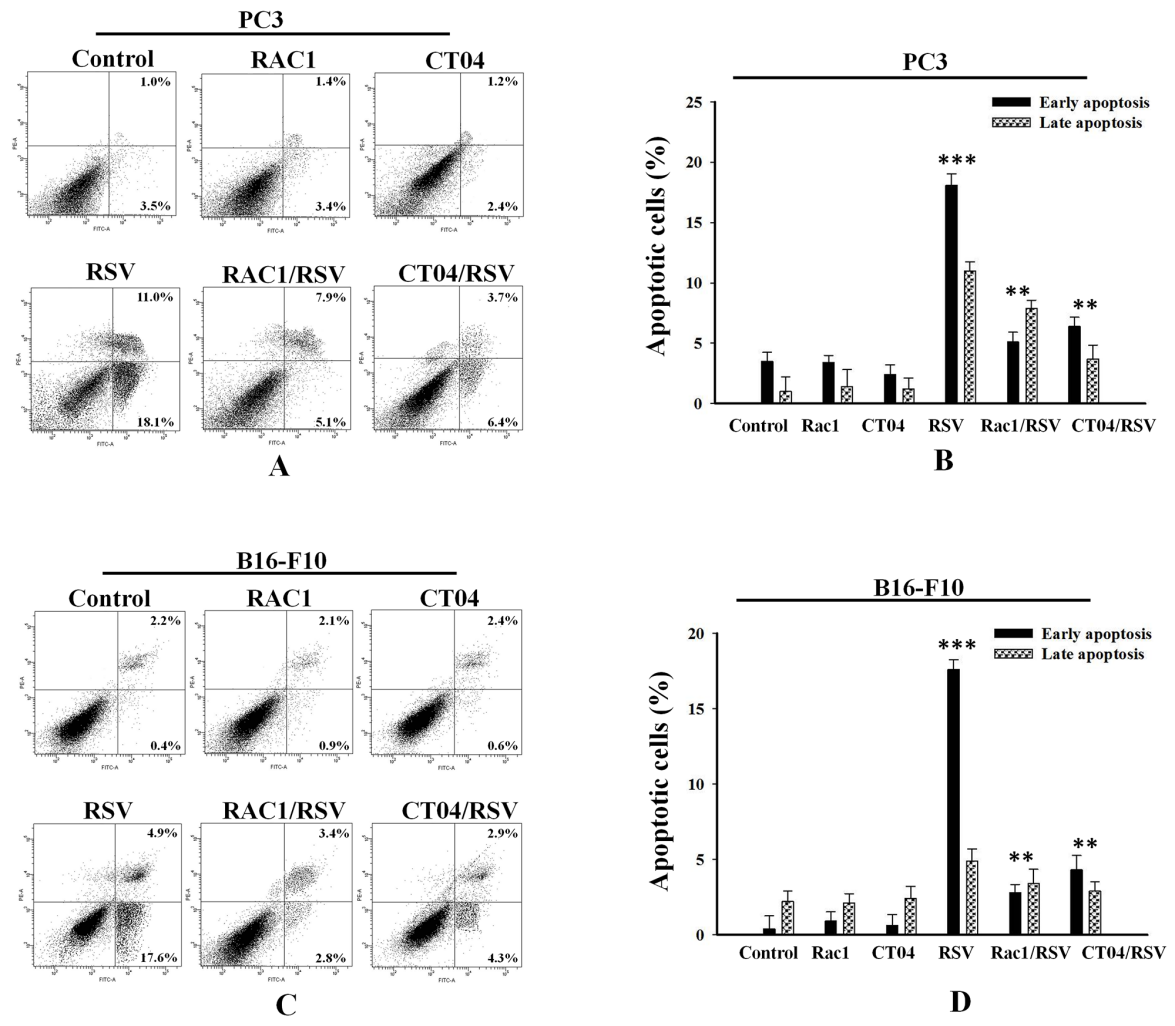
Modulation of Rho family proteins inhibits resveratrol-induced apoptosis in cancer cell lines Annexin-V/PI apoptosis assay in PC3 **(A, B)** and B16-F10 **(C, D)** cells transfected with Rac1 or treated with the RhoA inhibitor CT04, and incubated with or without 10 μM RSV. Lower and upper right panels are included in the analysis, and data is presented as means ± SD. **P < 0.01; ***P < 0.001.

To confirm that PRPF is responsible for blocking resveratrol-induced apoptosis by regulating Rac1 and RhoA, an annexin V/PI assay was carried out in PC3 prostate cancer, and B16-F10 melanoma cells. In PC3 cells, Rac1 overexpression decreased the percentage of cells in early apoptosis to 5.1% and in late apoptosis to 7.9%, compared to the percentages of resveratrol-treated PC3 cells in early and late apoptosis, at 18.10% and 11%, respectively (Figure 4A, B). Likewise, CT04 treatment reduced the percentage of cells in early apoptosis to 6.4% and in late apoptosis to 3.7% (Figure 4A, B). In B16-F10 melanoma cells, both Rac1 transfection and CT04 treatment reduced the percentages of cells in early and late apoptosis to 2.8% and 3.8%, for Rac1-transfected cells, and to 4.3% and 2.9%, for CT04-treated cells, respectively (Figure 4C, D). Additionally, we confirmed the regulatory effect of Rho family proteins on the rearrangements of the actin cytoskeleton in melanoma cells through confocal microscopy. Compared to untreated cells, Rac1-overexpressing B16-F10 cells showed a decreased number of stress fibers, and induced lamellipodia-like projections (Supplemental Figure 1B). Treatment with the Rho inhibitor CT04 also diminished the number of stress fibers and induced morphological changes (Supplemental Figure 1B). These findings suggest that PRPF may inhibit resveratrol-induced apoptosis in cancer cells by regulating Rho family proteins.

### PRPF inhibits cell invasion and may promote anticancer drug resistance by inducing EMT

**Figure 5:**
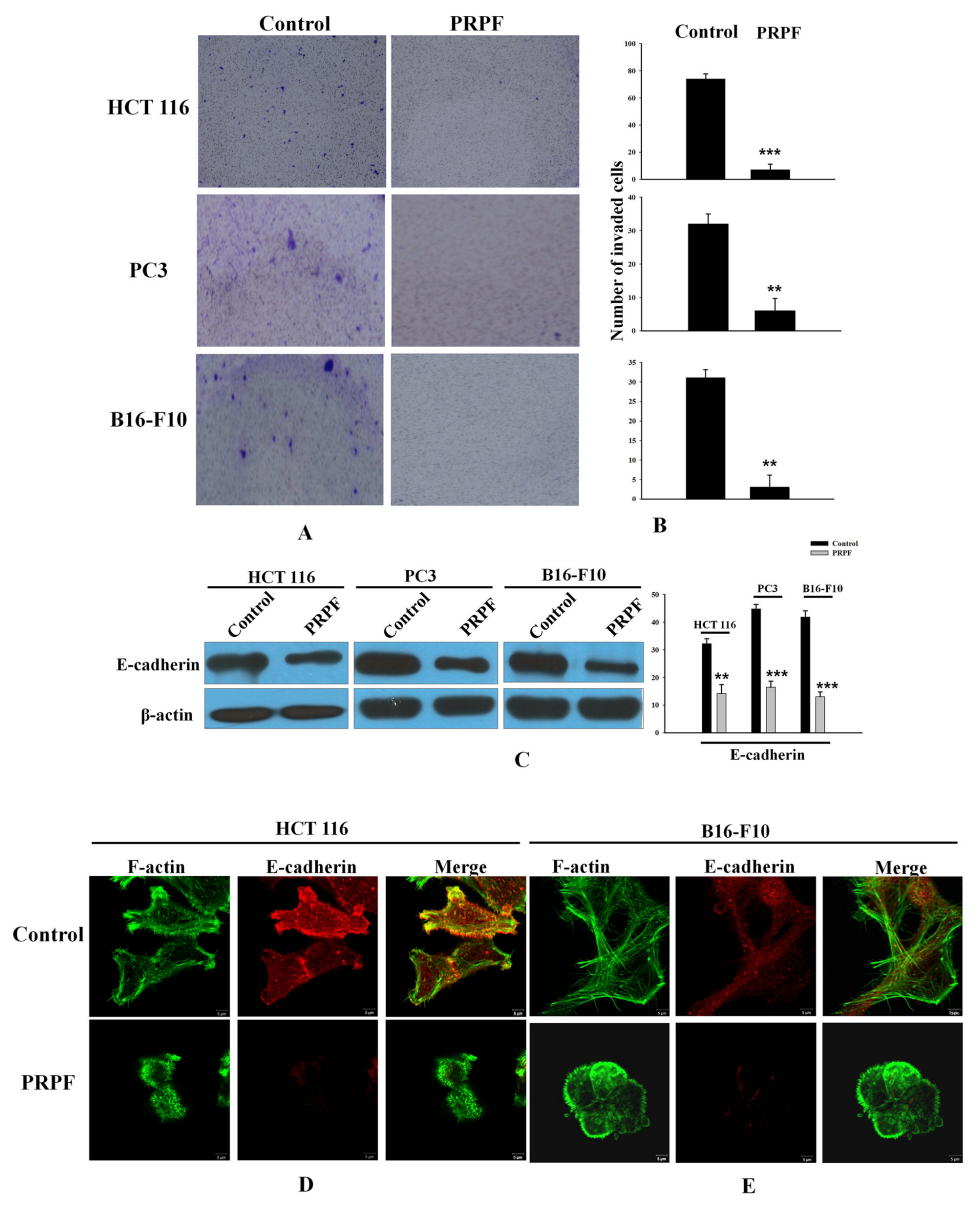
PRPF inhibits the invasion of cancer cells by inducing EMT **(A, B)** Cellular invasion assay. Blue spots represent invaded cells. Representative images and their quantifications are shown. The experiment was repeated three times, and data represent the means ± SD. **(C)** E-cadherin protein levels following PRPF overexpression, determined by western blotting. Actin is used as an internal control. **(D, E)** Immunofluorescence microscopy indicating the expression and localization of F-actin and E-cadherin following PRPF overexpression in HCT116 and B16-F10 cells. Cells are immunostained with phalloidin and anti-E-cadherin antibodies. The colocalization of the phalloidin and E-cadherin staining is shown in the merged images.

In control HCT116 cells, F-actin filaments were scattered throughout the cytoplasm, a pattern representative of the typical organization in migrating cells. However, in PRPF-transfected, Rac1-transfected, and CT04-treated cells, confocal microscopy images showed that F-actin was differently distributed. Malignant cells undergoing EMT display increased motility, acquire a mesenchymal phenotype, and finally become highly invasive. To examine typical EMT behavior, we analyzed the cellular invasiveness of control and PRPF-transfected HCT116 cells with a Boyden Millipore chamber system, and found that PRPF transfection of HCT116 cells led to a substantial (10.57-fold) decrease in cellular invasiveness (Figure 5A, B). Next, to examine the impact of PRPF overexpression on cell invasion in other cancer cell lines, we conducted cell migration assays using PC3 and B16-F10 cells, and observed that PRPF transfection consistently reduced invasion of both cell lines by 5.33- and 10.33-fold, respectively (Figure 5A, B).

It has been suggested that the downregulation of intracellular adhesion molecules disrupts cell-cell adhesion, which is specific to EMT [27]. E-cadherin downregulation is considered one of the most reliable markers of EMT [28]. We investigated the effect of PRPF overexpression on E-cadherin protein levels by western blotting, and found that PRPF transfection effectively decreased E-cadherin expression in HCT116, PC3, and B16-F10 cancer cells (Figure 5C). To confirm these observations, we evaluated the structural changes in the actin cytoskeleton and E-cadherin by immunostaining in HCT116 and B16-F10 cells. In PRPF-overexpressing HCT116 and B16-F10 cells, the number of stress fibers was significantly reduced compared to the number in control cancer cells (Figure 5D, E). Interestingly, PRPF transfection in both cancer cell lines downregulated E-cadherin protein levels, concomitant with an apparent loss of cell-cell junctions (Figure 5D, E).

**Figure 6:**
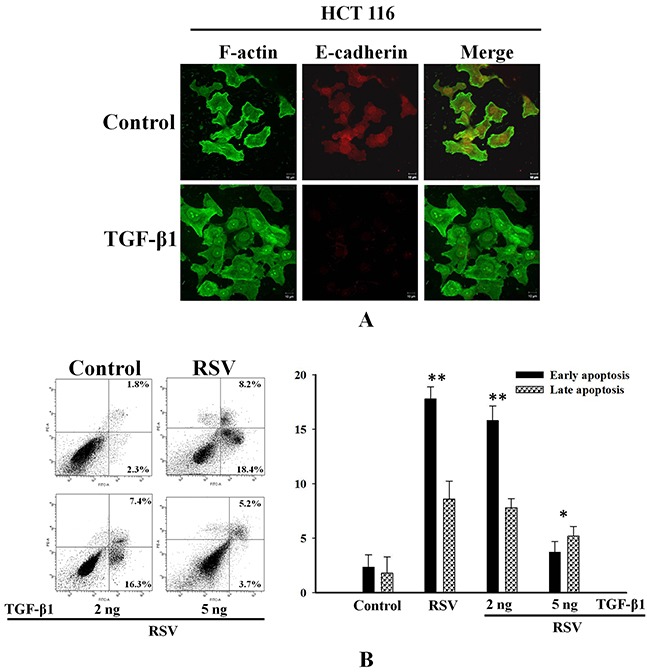
TGF-β1 inhibits resveratrol-induced apoptosis through the induction of EMT in HCT116 cells **(A)** HCT116 cells pretreated with 2 or 5 ng of TGF-β1, and incubated with RSV. Cells are double-stained for F-actin and E-cadherin and analyzed by confocal microscopy. **(B)** Inhibitory effect of TGF-β1 against resveratrol-induced apoptosis using an annexin-V/PI apoptosis assay. Data represent the means ± SD. *P < 0.05; ***P < 0.01.

Transforming growth factor-β (TGF-β) has been reported to be a potent inducer of EMT in cancer cells [29–30]. To investigate the relationship between EMT and drug resistance in HCT116 cells, we analyzed the effect of TGF-β1 on changes in the actin cytoskeleton and the expression of E-cadherin. TGF-β1-treated cells showed fewer stress fibers and considerably lower E-cadherin levels compared to those in untreated cells (Figure 6A). To confirm the inhibition of resveratrol-induced apoptosis, we conducted an annexin V/PI assay. Treatment with low levels (2 ng) of TGF-β1 had a negligible effect on apoptosis, whereas treatment with 5 ng of TGF-β1 significantly reduced the percentage of cells in early and late resveratrol-induced apoptosis to 3.7% and 5.2%, respectively (Figure 6B). Collectively, these data suggest that PRPF may induce the EMT, thereby leading to resistance against resveratrol-induced apoptosis.

## DISCUSSION

The role of PRPF kinase in the control of cell growth in human cancer cell lines has been well documented. For example, in an siRNA-based screen for kinases involved in pancreatic cancer cell survival, knockdown of PRPF was shown to increase apoptosis and decrease cell viability [30]. Structural approaches provided evidence that the kinase domain of PRPF was essential for regulating cancer cell growth and survival [7]. We previously reported that PRPF enhanced antioxidant enzyme activation in human colon cancer and mouse melanoma cells, which suppressed the generation of ROS and inhibited cell death [20]. PRPF kinase was first shown to be essential for pre-mRNA splicing. A network-based analysis of colon cancer splicing changes reported noteworthy differences in the induction of various transcription factors such as MYC and ELK1 [11], and supported our findings with respect to the activation of signaling pathways upon PRPF overexpression (Figure 2B). ELK1 transcription is induced by mutant k-RAS through the RAS-MAPK pathway [11], and that is consistent with our observation of an increase in the activity of the RAS-ERK pathway following PRPF overexpression (Figure 2B). PRPF overexpression may induce changes in splicing patterns, rescuing cell death, partly by inhibiting the generation of resveratrol-induced ROS, and regulating various cell survival signaling pathways (Figures 1C, D and 2A).

In addition, PRPF overexpression induced morphological changes by modulating Rho family proteins such as RhoA and Rac1. PRPF overexpression rearranged the actin cytoskeleton in HCT116 cells, and conferred resistance to resveratrol-induced cell death. Rac1 knockdown with siRNA can enhance drug-induced growth inhibition, cell cycle arrest, and apoptosis [32]. The results of the present study suggest that resistance to resveratrol-induced cell death conferred by PRPF overexpression may be mediated through the modulation of the actin cytoskeleton, which is regulated by RhoA and Rac1 (Figure 3). These results indicated that RhoA activation and Rac1 upregulation are probably correlated with actin cytoskeleton rearrangements and a suppression of cellular invasiveness. Rac1 and EMT are associated with invasive cancers and chemotherapy resistance. EMT is a complex cellular program by which epithelial cells acquire a mesenchymal-like phenotype by losing their epithelial characteristics. Although EMT plays a critical role in drug resistance in cancer, the exact mechanism is unclear [15, 33]. As shown in Figure 5, upon PRPF overexpression, HCT116 cells became rounded and detached from each other. It seemed that these cells had lost their epithelial characteristics and acquired a mesenchymal-like phenotype. Loss of E-cadherin is considered to be one of the most reliable markers of EMT, and we found that PRPF downregulated the expression of E-cadherin in HCT116, PC3, and B16-F10 cells (Figure 5). Several studies have shown that TGF-β signaling plays an important role in EMT. Treatment with TGF-β is one of the easiest ways to induce EMT in various epithelial cell lines [30]. TGF-β1 effectively downregulated E-cadherin protein levels in HCT116 cells, and inhibited resveratrol-induced cell death (Figure 6). These findings imply that PRPF may confer drug resistance to resveratrol, partly by driving cancer cells toward EMT. We postulate here that PRPF, through its potent mRNA splicing regulatory activity, alters the expression of EMT-associated genes in HCT116 cells to promote this transition. Moreover, it affects the expression of various other yet unknown proteins that are involved in increasing drug resistance and reducing cell invasion.

**Figure 7:**
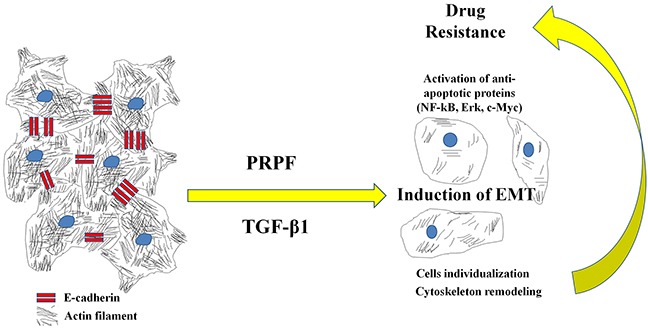
Graphical abstract Proposed model for the action of PRPF overexpression.

Based on the results of our study, we conclude that PRPF promotes drug resistance to resveratrol and blocks cell death by inhibiting the resveratrol-induced generation of ROS, by remodeling the actin cytoskeleton, activating cell survival signaling pathways, and inducing EMT (Figure 7. Graphical abstract). The further evaluation of PRPF-induced anticancer drug resistance and anti-apoptotic mechanisms may lead to novel approaches for overcoming drug resistance in patients with cancer.

## MATERIALS AND METHODS

### Chemicals and reagents

Cell lines were obtained from American Type Culture Collection (Manassas, VA, USA). RPMI 1640, Dulbecco's modified Eagle's medium (DMEM), fetal bovine serum (FBS), and penicillin/streptomycin were purchased from Gibco (Carlsbad, CA, USA). Alexa Fluor 488 Phalloidin and 2′,7′-dichlorofluorescein diacetate (H2DCFDA) were purchased from Invitrogen (Eugene, OR, USA). The Annexin-V-FITC Apoptosis Detection Kit was obtained from Abcam (Cambridge, UK). The Rho Activation Assay Biochem Kit (BK036-S) and CT04 were purchased from Cytoskeleton, Inc. (Denver, CO, USA). ECM Invasion Chambers were obtained from Merck Millipore (Temecula, CA, USA). PRPF and Rac1 cDNA clones were purchased from Sino Biological Inc. (North Wales, PA, USA), and PRPF siRNA was obtained from Santa Cruz Biotechnology (SC-76257; Santa Cruz, CA, USA). Resveratrol was purchased from Santa Cruz Biotechnology (CAS 501-36-0). N-Acetyl cysteine (NAC) was obtained from Sigma-Aldrich (St. Louis, MO, USA). SuperScript III Reverse Transcriptase was purchased from Invitrogen. Xfect Transfection Reagent was obtained from Takara Bio USA, Inc. Lipofectamine RNAiMAX Transfection Reagent was purchased from Invitrogen. Antibodies were obtained from Santa Cruz Biotech [PRPF (s-130856), β-actin (sc-47778), Rac1 (sc-95), NFκB p52 (sc-7386), IκBα (sc-1643), ERK (sc-292838), p-ERK (sc-7383), JNK (sc-7345), p-JNK (sc-6254), and c-MYC (sc-40)] and Cell Signaling Technology [AKT (cat# 9272), p-AKT (cat# 9271), E-cadherin (cat# 3195), and RhoA (cat# 2117)].

### Cell culture

HCT116 (ATCC CCL-247), PC-3 (ATCC CRL-1435), and B16-F10 (ATCC CRL-6475) cells were cultured in DMEM, and HCT-15 (ATCC CCL-225) cells were cultured in RPMI-1640 medium. Both mediums were supplemented with 10% FBS, and 1% penicillin-streptomycin, and cells were maintained at 37°C in a humidified atmosphere containing 5% CO_2_.

### Measurement of ROS generation

We measured ROS as previously described, with a slight modification [22]. Briefly, cells were seeded on 12-mm microscope coverslips in 24-well plates (2 × 10^5^ cells/well). At 80 % confluence, cells were transfected with a PRPF expression plasmid and incubated with or without resveratrol for the indicated time. NAC was used as a reference. The medium was replaced with fresh full growth DMEM/RPMI-1640 medium, and cells were incubated with the DCFHDA probe for 30 min. Then, the cells were washed twice with ice-cold PBS, and coverslips were carefully placed on 25 mm × 75 mm microscopic slides (S8902, Sigma Aldrich). Images were captured with a confocal laser scanning platform (DM/R-TCS, Leica) coupled to a microscope (Leitz DM REB), at excitation and emission wavelengths of 480 and 520 nm, respectively.

### Annexin-V-FITC apoptosis assay

An apoptosis assay was performed according to the manufacturer's instructions (Abcam: ab14085). Briefly, in one set of experiments, cells were seeded in 6-well plates (2 × 10^5^ cells/well), and transfected with a PRPF or Rac1 plasmid and/or incubated with resveratrol. In another experiment, cells were pre-treated with CT04 (1 μg/mL) and then incubated with or without resveratrol for 24 h. Next, cells were gently trypsinized, collected by centrifugation, and resuspended in 500 μL of 1X binding buffer. Finally, the cells were incubated with annexin-V-FITC and propidium iodide (PI: 50 mg/mL; 5 μL each) at room temperature for 5 min in the dark, and analyzed by flow cytometry (FACSARIA III; BD Biosciences, USA).

### Immunofluorescence microscopy

Alexa Fluor 488 phalloidin (1:40 dilution; Invitrogen Molecular Probes, Eugene, OR, USA) was used for F-actin visualization. Briefly, the cells were washed twice with PBS and fixed with 3.7% formaldehyde for 10 min at room temperature. Cells were then permeabilized with 0.1% Triton X-100 for 4 min, and washed twice with PBS. An Alexa Fluor 488 phalloidin working solution (6.6 μM in methanol), diluted with 500 μL of 2% BSA, was added to the cells and incubated for 20 min at 25°C in the dark. Finally, the cells were analyzed using confocal microscopy.

### Plasmid transfection and gene knock down by siRNA

Rac1 and PRPF overexpression was achieved by transfecting cells with Rac1 and PRPF expression plasmids, respectively. Briefly, HCT116 cells were cultured at a density of 1 × 10^6^ cells to 50% confluence. Then, the PRPF plasmid was transfected into the cells using the Xfect Transfection Reagent, whereas siRNA-PRPF was transfected with RNAiMAX, according to the manufacturer's instructions. Gene overexpression and downregulation were confirmed by agarose gel electrophoresis and SDS-PAGE.

### Cignal 45-Pathway Reporter Array

Forty-five signaling pathways were analyzed with the Signal Transduction Reporter Array (Qiagen, CCA-901L) according to the manufacturer's protocol. Briefly, HCT116 cells were cultured in 6-well plates and transfected with PRPF or scramble control for the indicated period, after which they were plated on the Cignal Signal Transduction Reporter Array for transfection with a mixture of an inducible transcription factor responsive firefly luciferase reporter. Luciferase activity was measured using the Dual Luciferase Assay system (Promega) on a Glomax multi luminometer (Promega). Firefly luciferase served as the experimental reporter, and Renilla luciferase was used for normalization.

### Rho activation assay

RhoA activation was analyzed with a glutathione S-transferase (GST) pull-down assay (Cytoskeleton, Inc. 1830 S Acoma St, Denver, CO 80223) according to the manufacturer's instructions. Activated RhoA was pulled down using a GST fusion protein containing the Rho binding domain (RBD) of the Rho effector protein rhotekin. Briefly, cells were maintained in serum-free medium for 24 h to silence endogenous activation of Rho GTPases. Then, the cells were transfected with PRPF, and supplemented with medium to reactivate Rho GTPases. After 30 min of activation, the cells were lysed, and total cell lysates were incubated with the GST-RBD fusion protein overnight at 4°C. The reactivated Rho GTPase bound to the GST fusion protein in the lysate was pulled down with colored glutathione-sepharose beads. Rho GTPase was detected by immunoblotting using a mouse anti-RhoA antibody. Total RhoA in primary lysates was detected in parallel.

### Boyden chamber invasion assay

The impact of PRPF on HCT116 cell invasion was investigated using the Boyden Millipore chamber system. Briefly, inserts were incubated in serum-free medium for 2 h at room temperature to rehydrate the ECM layer. Then, cells were seeded onto the membrane of the upper chamber insert, and placed in the wells of a 24-well plate containing 500 μL of full growth medium, and transfected with PRPF. After the indicated times, the non-invading cells remaining on the upper surface of the membrane were removed with cotton swabs. The cells that had invaded across the collagen to the lower surface of the membrane were stained with 5% Giemsa solution for 20 min at room temperature in the dark, for visualization. Cells were washed with PBS, air-dried, and images were captured using a Nikon SMZ18 system.

### Reverse transcription-polymerase chain reaction (RT-PCR)

Total RNA was extracted from cells with TRIzol reagent (Invitrogen), and 5 μg of the total RNA was reverse transcribed using SuperScript III Reverse Transcriptase according to the manufacturer's protocol. The resulting cDNA was incubated with RNase H at 37°C for 2 h, and PCR was performed with cDNA (2 μL) and the following primers: PRPF forward, 5′-AGGGATCGAAGCTGGAAATA-3′ and PRPF reverse, 5′-TGACCTCTGAGTCATCT-GTGG-3′; GAPDH for-ward, 5′-AGGGCTGCTTTTA-ACTCTGGT-3′ and GAPDH reverse, 5′-CCCCACTTGATTTTGGAGGGA-3′. PCR was performed under the following conditions: one cycle at 98°C for 3 min, followed by 30–40 cycles at 95°C for 30 sec, 55°C for 30 sec, and 72°C for 30 sec, with a final extension step at 72°C for 5 min. The amplified PCR products were analyzed by 2% agarose gel electrophoresis with EcoDye™ Nucleic Acid Staining Solution (Biofact Co., Ltd.), and the relative intensities of the detected bands were measured on a Gel Doc2000 scanner (Bio-Rad, Hercules, CA, USA).

### Western blot

Cell lysates were prepared using cell lysis buffer (50 mM Tris, pH 7.4, 0.5% NP40, 0.01% sodium dodecyl sulfate [SDS], and protease inhibitor cocktail [Roche, Germany]), and the protein concentration was determined using the Bio-Rad Protein Assay according to the manufacturer's protocol. Samples (30 μg) were prepared in SDS sample buffer containing 60 mM Tris-HCl (pH 6.8), 2% SDS, 10% glycerol, and 5% β-mercaptoethanol, separated on a 10% SDS-PAGE gel, and transferred onto a polyvinylidene fluoride (PVDF) membrane (Amersham, Piscataway, NJ, USA). The membranes were blocked with 3% albumin (Gendept, USA) solution for 1 h at room temperature. Chemiluminescent signals were developed with Clarity™ ECL Western Blotting Substrate (Bio-Rad) according to the manufacturer's instructions.

### Statistical analysis

All samples were prepared in triplicate, and all experiments were repeated at least three times. The data represent the means ± SD. Differences between groups were evaluated with the Student's t-test. P values less than 0.05 were considered statistically significant.

## SUPPLEMENTARY MATERIALS FIGURE


