# Supplementary material for: PRPF overexpression induces drug resistance through actin cytoskeleton rearrangement and epithelial-mesenchymal transition
Source: Oncotarget. 2017 May 15;8(34):56659–71. doi: 10.18632/oncotarget.17855 (PMC5593591; doi:10.18632/oncotarget.17855)
Supplement: Supplementary file 1 [file oncotarget-08-56659-s001.pdf]

PRPF overexpression induces drug resistance through actin cytoskeleton rearrangement and epithelial-mesenchymal transition

SUPPLEMENTARY MATERIAL

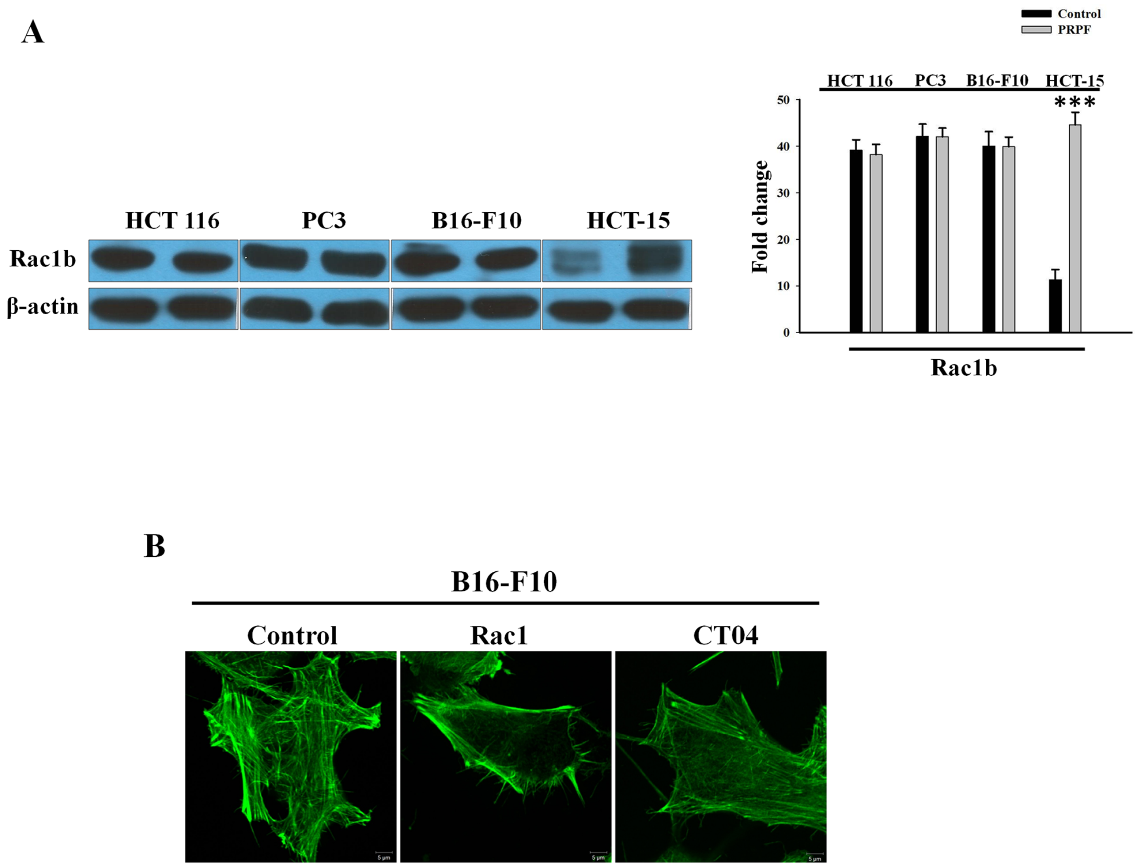

Supplementary Figure 1: The activity of PRPF is mediated by Rho family proteins. (A) Rac1B levels in total cell lysates from four cancer cell lines, analyzed by western blotting. Data represent the means±SD. ***P < 0.01. **(B)** Cytoskeletal dynamics in B16-F10 cells following Rac1 overexpression and RhoA inhibition. Cells are stained for F-actin, and analyzed by confocal microscopy at a magnification of 1000X.
